# Craving in Opioid Use Disorder: From Neurobiology to Clinical Practice

**DOI:** 10.3389/fpsyt.2019.00592

**Published:** 2019-08-30

**Authors:** Johan Kakko, Hannu Alho, Alexander Baldacchino, Rocío Molina, Felice Alfonso Nava, Gabriel Shaya

**Affiliations:** ^1^Department of Clinical Sciences, Psychiatry, Umeå University, Umeå, Sweden; ^2^Department of Public Health Solutions, The Alcohol, Drugs and Addictions Unit, National Institute of Health and Welfare, Helsinki, Finland; ^3^Division of Population and Behavioural Science, School of Medicine, University of St Andrews, St Andrews, United Kingdom; ^4^Centro de Atencion a las Adicciones de Arganzuela, Madrid Salud, Ayuntamiento de Madrid, Madrid, Spain; ^5^Director Penitentiary Medicine and Drug Abuse Unit, Health Care Unit Padua, Padua, Italy; ^6^Medical Affairs, Indivior UK Ltd, Slough, United Kingdom

**Keywords:** opioid, craving, addiction, negative affect, methadone, buprenorphine

## Abstract

Opioid use disorder (OUD) is a major public health issue that has reached epidemic levels in some parts of the world. It is a chronic and complex neurobiological disease associated with frequent relapse to drug taking. Craving, defined as an overwhelmingly strong desire or need to use a drug, is a central component of OUD and other substance use disorders. In this review, we describe the neurobiological and neuroendocrine pathways that underpin craving in OUD and also focus on the importance of assessing and treating craving in clinical practice. Craving is strongly associated with patients returning to opioid misuse and is therefore an important treatment target to reduce the risk of relapse and improve patients’ quality of life. Opioid agonist therapies (OAT), such as buprenorphine and methadone, can significantly reduce craving and relapse risk, and it is essential that patients are treated optimally with these therapies. There is also evidence to support the benefits of non-pharmacological approaches, such as cognitive behavioral therapy and mindfulness-based interventions, as supplementary treatments to opioid agonist therapies. However, despite the positive impact of these treatments on craving, many OUD patients continue to suffer with negative affect and dysphoria. There is a clear need for further studies to progress our understanding of the neurobiological basis of craving and addiction and to identify novel therapeutic strategies as well as to optimize the use of existing treatments to improve outcomes for the growing numbers of patients affected by OUD.

## Introduction

Opioid use disorder (OUD) is a major public health burden worldwide and is associated with substantial mortality and morbidity (1, 2). A key component of OUD is the development of long-lasting drug craving (3–5), whether in the context of prescribed opioids, such as for analgesic purposes, or illicitly acquired heroin. Although a universally agreed definition of drug craving is lacking, in the literature, it has been defined as an intrusive and overwhelming strong desire or compulsion to use a drug because of the memory of the pleasant rewarding effects superimposed on a negative emotional state (6–8). Craving is considered to be central to the motivational drive in addiction (9) and is recognized as an integral component of dependence syndromes, as acknowledged by its incorporation within the latest Diagnostic and Statistical Manual of Mental Disorders 5 classification system for substance use disorders (10). Craving is also included within the definition of opioid dependence in the recently updated 11th Revision of the International Classification of Diseases system (11).

Craving is a common symptom across substance use disorders beyond opioids, including those relating to alcohol, nicotine, cannabis, cocaine, and other psychoactive substances (11). Although there have been several articles in recent years reviewing data in drug craving (9, 12), a focused review of opioid-specific evidence is lacking. In this review, we will discuss the neurobiological foundations for craving, approaches to assessing craving, the importance of craving for predicting the risk of relapse in clinical practice, and the potential for therapies to target craving in OUD.

To identify relevant references, a narrative literature search was conducted in April 2019. The PubMed database was searched using terms related to opioids and craving. Only articles in English were included. Additional references were identified through searching the bibliographies of retrieved articles.

## Neurobiology of Addiction and Craving in Opioid Use Disorder

Addiction is a chronic relapsing disease with a neurological basis (13, 14). It is therefore important to explore the neurobiological underpinnings of craving in order to better understand the disease process and to identify potential targets of anti-craving medications and non-pharmacological interventions (15). A range of theories describing various aspects of neuroadaptation in addiction have been proposed, including opponent, inhibitory control, reward deficiency, incentive sensitization, aberrant learning, and anti-reward theory (16). The opponent theory, for example, proposes that the euphoria induced by a drug is opposed by a counteracting process that eventually masks the initial hedonic effects (17). In addition, the incentive sensitization theory has been proposed as being particularly relevant for craving. This theory suggests that repeated use of illicit drugs induces neuroadaptations leading to enduring sensitization of dopamine systems with subsequent hyperreactivity in response to drug cues and a pathological degree of incentive salience, manifesting as a disproportionate motivation to pursue the drug (16, 18, 19). Such an increase in salience associated with drug-related cues is coupled with a reduced sensitivity to normal, non-drug-related rewards (20). A detailed explanation of the various theories of addiction is beyond the scope of the present review and can be found elsewhere (16).

**Figure 1:**
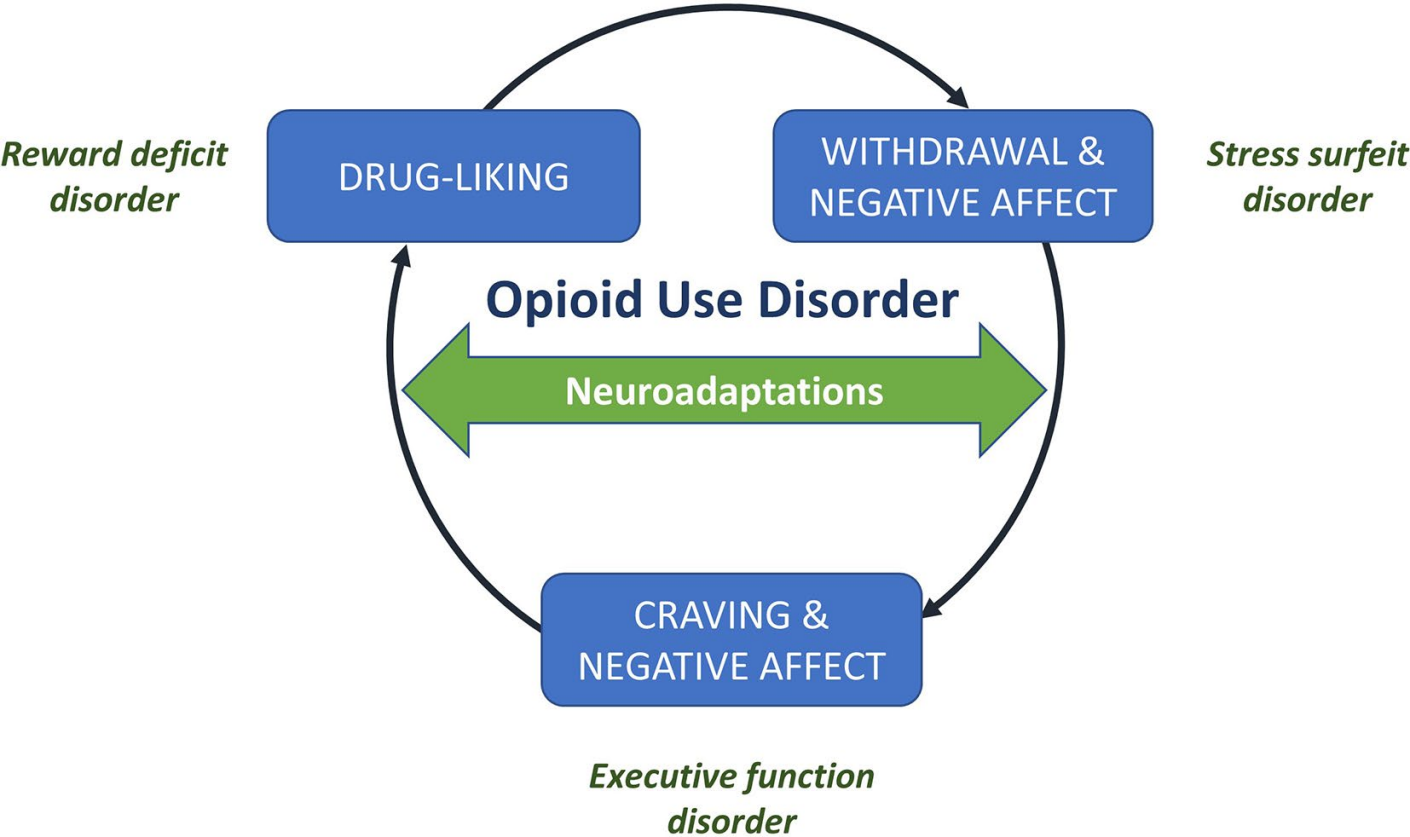
Drivers in the cycle of addiction. The addiction cycle involves three key drivers: (1) pleasurable drug-liking (associated with euphoria in the early stages of addiction), (2) withdrawal and negative affect (the stress and dysphoria associated with withdrawal from a drug), and (3) craving for the drug and ongoing negative affect. Underlying these key drivers are neuroadaptations associated with reward deficit, stress surfeit, and executive function disorders, respectively. Figure reproduced and adapted under the CC-BY license from the ACH Servier Research Group (22).

Although early models of addiction focused on hedonic reward, there is now evidence that negative reinforcement may have a particularly important role in maintaining addictive behaviors (21). In this review, we focus our discussion on the neurobiology of addiction based on the cycle of addiction developed by Koob and colleagues (22) (**Figure 1**).

**Figure 2:**
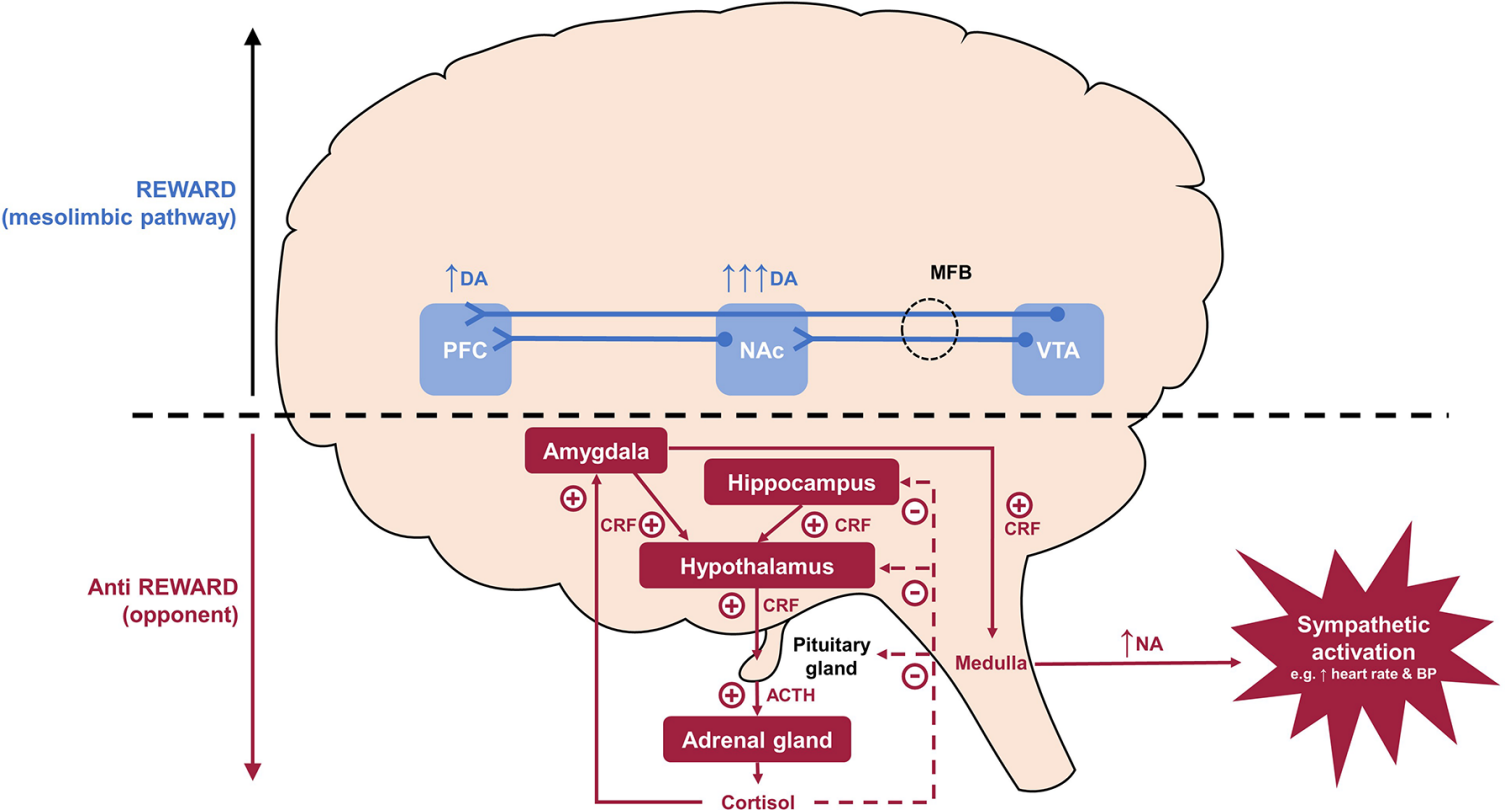
Interface between addiction and stress: Reward versus anti-reward. During acute drug use, the reward system becomes overactive and dopamine is upregulated, especially in the NAc, giving rise to positive reinforcing symptoms, such as drug-liking and euphoria. In the dependent state, the reward system becomes down-regulated and the anti-reward system becomes upregulated and CRF released from the hypothalamic neurons acts on the pituitary to release ACTH, which in turn results in the secretion of cortisol by the adrenal glands. The production of CRF is initially controlled by negative feedback of cortisol on the hippocampus and hypothalamus. However, this is eventually overcome by the production of *extra-hypothalamic* CRF from the amygdala in a feed-forward manner, which maintains the sympathetic nervous system stress response. This latter pathway produces a stress surfeit that contributes to the negative emotions associated with withdrawal and which goes unbuffered because of the hypodopaminergic tone in the mesolimbic pathway. ACTH, adrenocorticotropic hormone; BP, blood pressure; CRF, corticotropin-releasing factor; DA, dopamine; MFB, medial forebrain bundle; NA, noradrenaline; NAc, nucleus accumbens; PFC, prefrontal cortex; VTA, ventral tegmental area.

**Figure 3:**
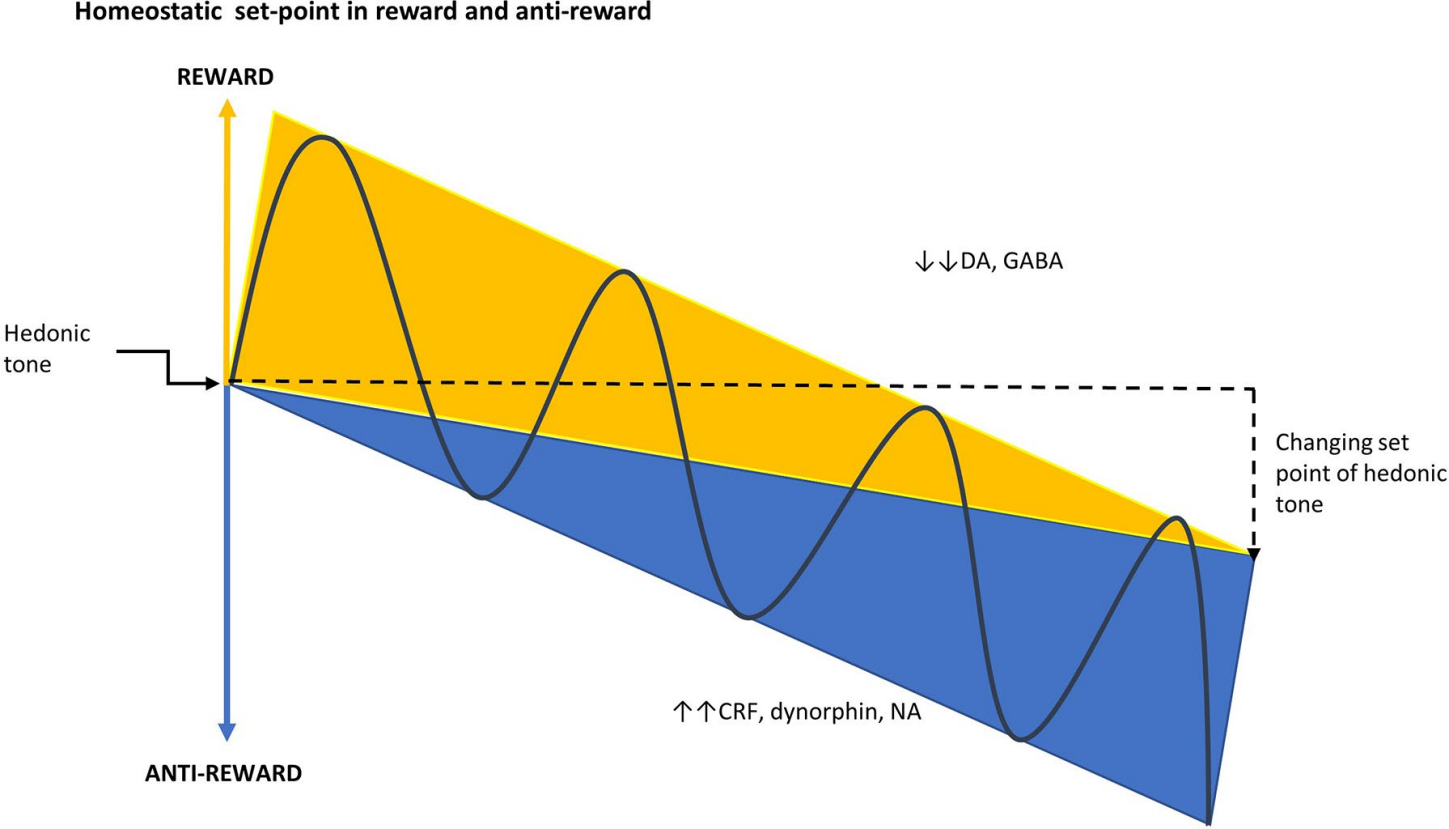
Homeostatic set-point in reward and anti-reward. The set-point of hedonic tone is determined by the balance between the opposing reward and anti-reward pathways. As addiction develops, there is a change in hedonic tone resulting from the deviation of the set-point that occurs when the reward system is down-regulated and the anti-reward system becomes dominant (25). CRF, corticotropin-releasing factor; DA, dopamine; GABA, γ-aminobutyric acid; NA, noradrenaline.

The cycle of addiction has been proposed to involve three key drivers that underlie the neurobiological changes associated with opioid dependence: i) drug-liking (due to drug intoxication), of which the positive emotions, such as euphoria, are positively reinforcing and increase the probability of using the drug in the early stage of addiction; ii) withdrawal and negative affect, which is associated with negative reinforcement because of the desire to consume a drug in order to improve the affective state and to offset the withdrawal symptoms; and, iii) craving, which relates to the intrusive preoccupation of wanting to use a drug, which can also be associated with negative affect (23, 24). All psychoactive drugs target the reward system in the brain producing the positive feelings and emotions that are associated with addiction or dependence (24). This system is opposed by the anti-reward system, which is responsible for the stress, dysphoria, and craving associated with withdrawal (**Figure 2**) (14). Addiction occurs when there is a deviation from the homeostatic set-point created by the reward and anti-reward systems in the brain (**Figure 3**) (25).

During acute intoxication, the mesolimbic pathway, comprising dopaminergic projections from the ventral tegmental area to the nucleus accumbens (NAc) and frontal cortex, becomes active and dopamine is upregulated, especially in the NAc. This gives rise to positive reinforcing symptoms, such as euphoria (hence the term reward pathway) (14, 24, 26). Other neurotransmitter systems implicated in positive reinforcement include endogenous opioids, γ-aminobutyric acid, glutamate, neuropeptide Y, and glucocorticoids (14). The frontal cortex function is relatively preserved in the early stages of addiction and is important in the subjective effects of craving, attribution of salience (assignment of relative value), and inhibitory behavioral control (24).

As addiction progresses, the anti-reward system is recruited through the extended amygdala [a macrostructure consisting of the NAc, amygdala, hypothalamus, and bed nucleus of the stria terminalis (27)] and activates stress pathways mediated by increases in dynorphins, corticotrophin-releasing factor (CRF), and noradrenaline (27). Dynorphins are opioid peptides that are widely distributed in the central nervous system and are increasingly recognized as key mediators of negative emotional states (27). In addiction, dynorphins are believed to play an important role in dysphoria, anhedonia, and compulsive drug-seeking behavior (27, 28). In response to acute intoxication, excess activation of dopamine receptors in the NAc can upregulate the production of dynorphins, which through kappa (κ)-opioid receptors (KOR), can exert negative feedback on dopamine release in the mesolimbic system and glutamate release in the NAc (27). This can lead to the mesolimbic system becoming hypodopaminergic and down-regulated, giving rise to anhedonia and tolerance to the euphoric effects of opioids (29).

The anti-reward system is also mediated by the release of CRF from the hypothalamus, which subsequently leads to the secretion of cortisol by the adrenal glands (27, 30). In the anti-reward system, the production of CRF is initially controlled by negative feedback at the level of the hypothalamus and pituitary; however, this brake may eventually be overcome by the production of *extra-hypothalamic* CRF from the amygdala, driven in a feed-forward manner by the secretion of cortisol. This drives the noradrenergic stress response (at the level of the brainstem) independently of the hypothalamic-pituitary-adrenal (HPA) axis. The release of dynorphins may also be under the control of CRF and conversely CRF release and/or function itself may be controlled by dynorphin-KOR signaling (31–33). A potential link between dynorphins and stress is also supported by the observation that KOR agonists have also been shown to increase cortisol levels in humans and monkeys (34, 35). Ultimately, this dominance of the anti-reward pathway may explain the negative emotions, such as malaise and dysphoria, experienced during withdrawal [reviewed in (14, 24)].

In OUD, the importance of the anti-reward system during the withdrawal state is supported by the evidence that cortisol levels are suppressed in patients with active opioid use (36–38), and then elevated in response to opioid withdrawal upon cessation (39–42). HPA axis dysregulation may persist well beyond the initial acute withdrawal phase, with one study demonstrating that salivary cortisol levels were significantly higher in OUD patients compared with controls for at least 25 days after detoxification (42). Furthermore, heroin craving has been shown to be positively correlated with serum cortisol levels [and negatively correlated with plasma β-endorphin levels (43)], emphasizing the need to target the anti-reward system in the long-term management of OUD. Exposure to drug-related cues mediates the symptoms of craving in OUD (44–49) and may be linked with increased cortisol responses (50), consistent with the neuroendocrine underpinnings of craving as described in **Figure 2**. Cue-induced drug seeking is also underpinned by the processes of conditioned reinforcement [described by Koob and Volkow (51) as a previously neutral stimulus that can reinforce behaviors through its association with a primary reinforcer and thus become a reinforcer in its own right] and incentive salience (defined as motivation for rewards that are derived from both a physiological state and previously learned associations about a reward cue). In this setting, activation of the dopamine system in response to repeated exposure to a reward leads to neuroadaptation, ultimately resulting in phasic dopamine system activation in response to neutral cues related to the reward, but not to the reward itself (22, 51).

Opioid agonist therapies (OAT), such as buprenorphine and methadone, have been shown to normalize the HPA axis (52, 53). However, despite the impact of OAT on the HPA axis, many heroin-dependent patients continue to experience negative affect, potentially as a result of signaling *via extra-hypothalamic* CRF (53). The significance of CRF in drug addiction has been extensively studied in animal models (27). Administration of a CRF receptor antagonist has been found to decrease opioid withdrawal-induced negative emotional states (54) and attenuated heroin self-administration in animals with extended access to the drug (55). Further to these preclinical findings, there has been growing interest in the therapeutic potential of selective CRF antagonists in addiction disorders and in depression, but to date, clinical trials have been unrewarding due to lack of efficacy or due to safety concerns (56). However, some preliminary evidence that CRF antagonism may reduce craving for heroin, albeit with no significant reduction in drug use, has been reported (57). Given the role of the dynorphin-KOR system in addiction, research efforts have also focused on the development of selective KOR antagonists (58, 59).

Over the past decade, functional imaging has provided further clarification of the relationship between craving and the neurobiological changes of addiction. For example, Li et al. (60) assessed neural responses among heroin-dependent patients following short-term heroin abstinence using functional brain imaging and found that increases in craving in response to drug-related cues were associated with activation of the mesolimbic dopamine pathway and the prefrontal cortex. Furthermore, similar functional imaging studies demonstrate involvement of the hippocampus and amygdala in cue-elicited craving (24), suggesting the importance of memories and emotion as substrates in craving.

## Clinical Assessment of Craving in Oud

Assessment of craving through effective patient-physician communication is crucial to the clinical management of patients with OUD. Furthermore, the use of simple assessment tools can provide further information on craving, which may be particularly valuable in the research setting as well as in clinical practice. A key challenge in developing reliable tools is the subjective nature of craving (61). Broadly, measures of craving can be divided into two categories—defined as observational and self-reported (61). Observational tools used previously in research studies rely on proxy measures, such as choosing access to drug over a monetary reward or willingness to work to access drug (61). Such tools avoid self-reporting bias and any communication challenges patients may face in articulating their craving symptoms (61). Such assessments are also less dependent on the conscious experience of craving and instead may capture unconscious craving impulses (61). However, observational methods can be challenging to implement in clinical practice and may reflect more than just craving alone (61, 62).

Self-reported craving can be evaluated by tools that are specific to craving or as a single item within a broader assessment tool (summarized in **Table 1**) (63, 72). In their simplest form, craving assessment tools can be single-item scales, which allow the responders to indicate their degree of craving using easy-to-understand Likert ratings or visual analogue scales (VAS) (61). Alternatively, multi-item scales can be used to assess craving (62), for example, the Opioid Craving Scale includes three questions each rated on a 0–10 VAS. The first question asks “How much do you currently crave opiates?,” while the remaining questions relate to cue-induced craving and likelihood of use in a prior drug-using environment (63). Self-reported assessments can be limited by ceiling and floor effects, that is, the scale imposes artificial limitations at either extreme, which may prevent distinguishing true differences in craving between patients (12). Although multi-item scales may offer improved reliability and sensitivity compared with single-item scales (12, 62), they can be cumbersome, time-consuming, and lead to respondent disengagement, demotivation, and increased reactivity (62, 63).

**Table 1:** Self-reported craving tools for opioid use disorders.

Tool	Number of items	Scale	Development
Opioid Craving Scale (63)	3 items	0–100 mm VAS for each item (total score calculated by averaging the scores on the 3 items)	Modified from the Cocaine Craving Scale
Heroin Craving Questionnaire (61, 64–66)	14- and 45-item versions available (45 item version includes five 9-item subscales)	7-point Likert scale for each item (total score calculated from individual items)	Modified from the Cocaine Craving Questionnaire
Modified Penn Alcohol Craving Scale (67)	5 items	7-point scale (total score calculated as mean of 5-item scores)	Modified from the Penn Alcohol Craving Scale
Desires for Drug Questionnaire (61, 67–69)	13 items within 3 domains (desire and intention, negative reinforcement, and control)	7-point Likert scale	Modified from the Desires for Alcohol Questionnaire
Cue-Elicited Craving Assessment (adapted for OUD) (46, 70)	1 item, administered after exposure to visual cues relating to opioid use	0–10 rating scale	OUD-focused version of generic cue reactivity test
Stress-Elicited Craving Assessment (adapted for OUD) (71)	1 item, administered after exposure to stress-inducing imagery	0–100 mm VAS scale	OUD-focused version of generic cue reactivity test
Screener and Opioid Assessments for Pain Patients revised version (72, 73)	24 items (1 item is specific to craving)	0–4 scale (item scores summed for total score)	Novel
Obsessive-Compulsive Drug Use Scale (61, 68, 69)	12 items within 3 domains (thoughts and interference, intention to use, and control of consumption)	5 choices per item	Modified from the Obsessive-Compulsive Drinking Scale

OUD, Opioid use disorder; VAS, Visual analogue scale.

In recent years, ecological momentary assessments (EMA) have emerged as a novel approach to assessing craving (74, 75). EMA involves patient recording of real-time assessments of craving using mobile technology at regular, random, or event-triggered (e.g., when the patient experiences craving) timepoints each day (74–77). Thus, EMA has the potential to enhance the understanding of craving, particularly in regard to temporal fluctuations (74, 75), and potentially to treatment dosing.

The US Food and Drug Administration (FDA) has issued guidance on patient-reported outcome instruments, such as self-reported craving assessments, specifying that instruments should meet a variety of criteria, including reliability, validity, specificity, and sensitivity (78). Furthermore, additional FDA guidance has emphasized that craving tools should complement other end points and correlate with a sustained clinical response (79).

## Craving in Clinical Practice

Some patients receiving maintenance treatment with buprenorphine or methadone continue to use illicit opioids, drop out from OAT treatment programs, or experience distressing craving symptoms (80). In addition, patients receiving opioid maintenance treatments frequently misuse other drugs, including alcohol, benzodiazepines, cannabis, and gabapentinoids (81, 82). In the European Quality Audit of Opioid Treatment (EQUATOR) survey of patients receiving OAT, 60% reported either regular or occasional use of illicit drugs while undergoing maintenance treatment, and 27% reported heroin use. Of patients who used illicit drugs in addition to OAT, one in six cited lack of control of cravings as the reason for illicit drug use (80). Furthermore, more serious psychiatric manifestations, such as suicidal ideation, depressed mood, and attempts to suppress distressing or intrusive thoughts, have been positively correlated with levels of self-reported opioid craving (83, 84),which is why craving symptoms should be monitored upon initiation of OAT.

Identifying and managing triggers for lapse (defined as a *temporary and controlled* return to drug use) and relapse (a *continuous and without control* return to drug use) remains an important part of the clinical management of OUD (85). Much research has focused on identifying predictors of relapse in substance use disorders, and one of the strongest candidates to emerge from these studies is craving (86). Although earlier studies provided inconsistent conclusions on the extent to which craving is associated with relapse (74), this may be due to the difficulty in measuring craving retrospectively using traditional recall methods (74). More recent studies utilizing EMA techniques have provided robust evidence that drug craving correlates with risk of subsequent opioid use both in patients receiving buprenorphine or methadone maintenance treatment and in the post-detoxification setting (74, 87–90). Furthermore, EMA methodology has demonstrated that the intensity of craving can increase linearly prior to drug use (87), and that the strength of opioid craving is positively associated with the severity of dependence and negatively associated with readiness to change drug use (91). Beyond the simple risk of relapse, the presence of drug craving can also be a distressing symptom and be disruptive to the functioning of patients (92).

To identify and manage the causes of craving, it may be useful to distinguish between background (tonic) and cue-induced (phasic) craving. Background craving is a slowly changing overall level of craving that occurs in the absence of external cues and may reflect the negative affect associated with abstinence or drug withdrawal and activation of the anti-reward system (9). In support of this theory, a correlation between negative affect and craving has been observed for many substance use disorders (74), and in OUD patients undergoing opioid withdrawal (93). In a study of patients with prescription-opioid dependence who had undergone medically-assisted withdrawal up to 14 days previously, patients with low levels of positive affect were found to be more vulnerable to craving on days when their positive affect was even lower than average (75).

In addition to background craving, episodes of fast-onset, relatively short duration spikes of craving (phasic craving) can be induced by specific drug cues or stressful life events (9), for which craving intensity correlates with stress severity (94). Of note, drug cue exposure and stress episodes can also have additive effects on drug craving (49). Measurements of the intensity, duration, and frequency of these craving episodes may all provide useful prognostic information regarding the risk of relapse (9). Furthermore, the identification of specific triggers for craving may also be a valuable therapeutic focus in the clinical consultation. EMA may allow greater understanding of both the tonic and phasic components of craving, which are typically challenging to differentiate outside of laboratory settings (9, 12, 74).

Even among heroin users who have been abstinent for greater than a year, drug-related cues still have the potential to elicit craving responses (47, 48), highlighting the enduring nature of craving among patients with a history of drug dependence. The influence of different cues on craving may also be dependent on the type of opioid dependence: in heroin dependence, cue-related craving was stimulated more intensely by paraphernalia images (e.g., needles), whereas patients with analgesic dependence were more stimulated by pills and pill bottles (46). The relationship between life stressors, craving, and relapse is complex and likely to be subject to significant inter-individual differences. For example, preliminary data suggest that women may experience greater increases in craving in response to stress than men (95).

Compared with drug cues, the relationship between pain and craving is less clear. In patients maintained on OAT, chronic pain was associated with three-fold higher odds of experiencing craving for opioids, although this did not lead to a significant increase in drug use (96). Conversely, in patients using prescription opioids for the treatment of chronic pain, craving was not found to be associated with pain itself, but rather with co-occurring anxiety and depressive symptoms, or with a perception of a magnified threat value of pain (97–99). For these patients, the intensity of craving is correlated with the risk of opioid misuse (70, 98, 100). Many of these patients may require treatment with OAT in order to improve outcomes (101).

## Craving as a Therapeutic Target in OUD

Inhibition of craving has long been a goal of OUD treatment (5). As early as the 1960s, experiments were being conducted using electric shock-based aversion therapy to suppress craving among patients addicted to synthetic opiates (102). In modern times, several pharmacological therapies, notably buprenorphine and methadone, are now available for the treatment of OUD (103). A comparison of the pharmacological characteristics and treatment regimens for methadone and buprenorphine is presented in **Table 2**. Methadone is a full μ-opioid receptor agonist, whereas buprenorphine is a high-affinity partial μ-opioid receptor agonist (103). Consequently, buprenorphine is associated with a ceiling effect with respect to the risk of respiratory depression (103). As a result of this high-affinity binding with buprenorphine, the effects from any additional illicit opioid use during maintenance treatment can be blocked (103). Other treatment options, including intravenous diamorphine, oral L-methadone, and slow-release oral morphine, have limited availability in most countries and are not considered in this review article.

**Table 2:** Comparison of oral/sublingual buprenorphine and methadone in OUD maintenance treatment.

	Buprenorphine	Methadone
Pharmacological targets	Partial µ-opioid receptor agonist (104) δ-opioid receptor antagonist (104) κ-opioid receptor antagonist (104) Opioid-like receptor-1 agonist (104)	Full µ-opioid receptor agonist (105) N-methyl-D-aspartate receptor antagonist (105)
Average half-life	32 h (106)	22 h (106)
Duration of induction	∼2–3 days (107)	2–4 weeks (“start low, go slow”) (7)
Typical induction regimen	2–8 mg on Day 1 (7) Each dose increased by 2–4 mg up to 24 mg daily (7)	≤30 mg/day (7) Increases of 5–10 mg over 5 or more days (7)
Typical maintenance dose	Maximum 24*mg daily (107)	60–120 mg daily (7)
Overdose risk	Low risk of overdose because of partial agonist effect and ceiling effect for respiratory depression (7)	Higher risk from overdose, particularly during induction (7)

*Higher maximum doses exist for some countries. Please consult local prescribing information. OUD, opioid use disorder.

**Figure 4:**
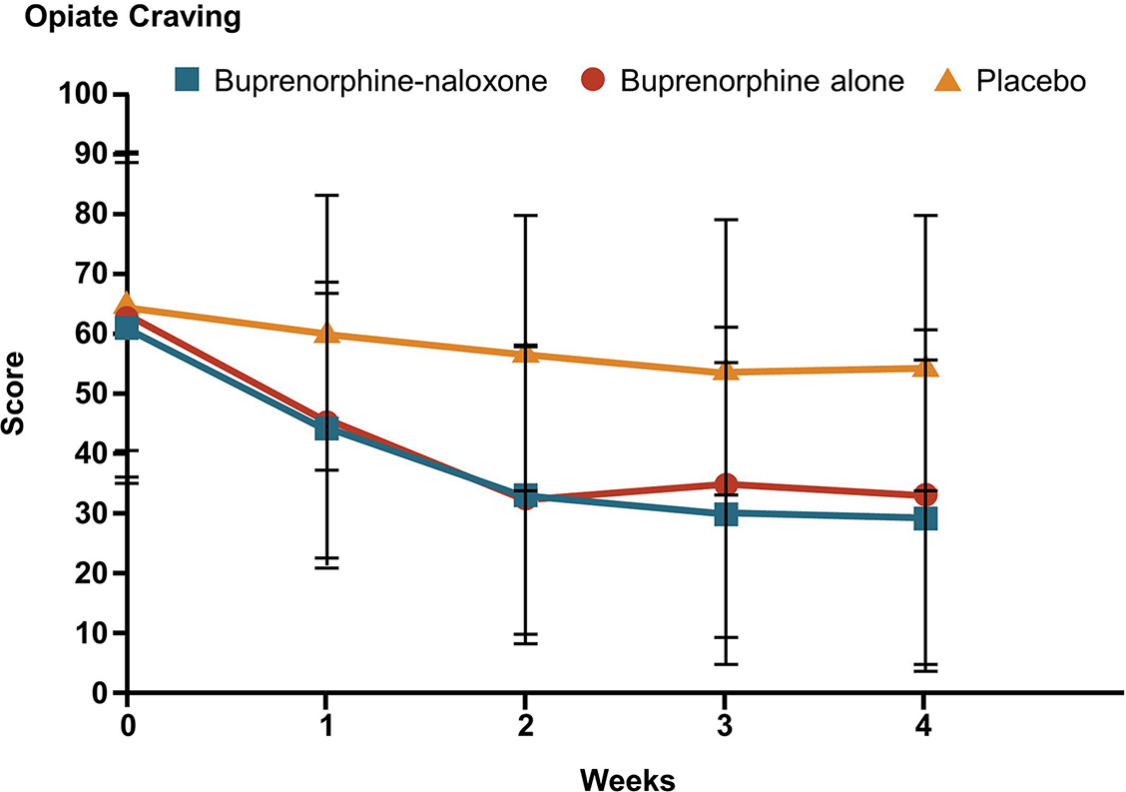
Impact of buprenorphine on self-reported opiate craving among OUD patients (111). Patients were randomized to double-blind treatment with sublingual tablets consisting of buprenorphine (16 mg), buprenorphine-naloxone (16 mg/4 mg), or placebo given daily for 4 weeks. Self-reported opiate craving was assessed as the peak craving during the prior 24 hours measured on a 0–100 mm visual analogue scale, with higher scores representing greater craving. Statistically significant reductions in craving (p < 0.001) were reported for comparisons between buprenorphine and buprenorphine-naloxone groups versus placebo at all post-baseline time points. OUD, opioid use disorder. Reproduced with permission from the Massachusetts Medical Society.

Numerous studies have demonstrated that buprenorphine and methadone can reduce craving in OUD patients (108, 109), which may explain why relapse rates are higher in patients who undergo detoxification or receive psychosocial interventions alone (105). Methadone was the first pharmacotherapy approved in OUD (7) and a systematic review performed by Fareed et al. (108) in 2011 identified seven studies that provided evidence supporting a role for methadone in reducing craving for heroin. This included a study by Shi et al. (110), which demonstrated that long-term methadone maintenance both improved mood and reduced cue-induced craving. A clear illustration of the impact of OAT on craving emerged from a randomized controlled trial comparing buprenorphine/naloxone formulation with placebo over 4 weeks in patients with opiate dependence. In this study, where craving was measured on a 0–100 mm VAS, mean craving more than halved, with a baseline score of 62.4 reducing to 29.8 after 4 weeks of treatment with a sublingual tablet formulation of 16 mg buprenorphine/4 mg naloxone (p < 0.0001, **Figure 4**) [(111) and unpublished data].

For both buprenorphine and methadone, the impact on craving is dose dependent (112, 113), and it is clear that in order to adequately suppress craving, buprenorphine and methadone must be carefully up-titrated to achieve the optimal dose for each patient (7). The relationship between buprenorphine dose and craving suppression is believed to be determined by the level of availability of μ-opioid receptors (114), and this has been explored in OUD patients using self-reported craving questionnaires and positron emission tomography with a μ-opioid receptor radioligand to explore receptor occupancy. In a preliminary study, Zubieta et al. (115) found that increasing buprenorphine dose {up to 16 mg given as a sublingual liquid [higher bioavailability compared to a sublingual tablet (116)]} was associated with a significantly greater suppression of craving scores and a 79–95% reduction in μ-opioid receptor availability with the 16 mg dose compared with placebo. This was corroborated by Greenwald et al. (117) who found that higher buprenorphine doses (up to 32 mg given as a tablet*) led to increased plasma buprenorphine levels, decreased μ-opioid receptor availability, and decreased withdrawal symptoms. In the same study, heroin craving was found to be significantly correlated with μ-opioid receptor availability and negatively correlated with buprenorphine plasma levels. These results were expanded in a further study exploring the chronology of μ-opioid receptor availability after buprenorphine dosing in heroin-dependent volunteers. In this study, craving intensity increased significantly from 4 to 76 hours post-dosing, correlating with increasing μ-opioid receptor availability from 30% to 82% over this time period among patients who had received buprenorphine (118).

In addition to its ability to reduce symptoms of withdrawal and craving in OUD, buprenorphine may also have a therapeutic impact on dysphoria (119), potentially leading to improvements in treatment retention and quality of life. The impact of buprenorphine on mood may relate to the antagonism it exhibits at κ-opioid receptors, as there is growing evidence that signaling through these receptors plays a role in the dysphoria and increased sensitivity to stress associated with the anti-reward pathway (120).

Substance use disorders can also be treated with the opioid antagonist naltrexone (121). However, in the context of OUD, naltrexone treatment may increase negative affect and craving through activation of the HPA axis by removal of tonic inhibition by endogenous opioids (122). Consistent with this, studies in OUD have demonstrated poor patient adherence to oral naltrexone and failed to show any therapeutic impact of this drug on craving (105, 109, 123, 124).

## Non-pharmacological Interventions

In substance use disorders, patients may benefit from a range of non-pharmacological interventions, such as cognitive behavioral therapy (CBT), motivational interventions, and contingency management (125). Motivational interventions/interviews typically involve brief sessions in which a counselor supports the patients in becoming motivated to change their drug use behavior (125, 126). Contingency management approaches provide direct rewards (e.g., vouchers, prizes, or special privileges) to the patients in response to a desired behavior (e.g., drug abstinence) (127).

CBT has been shown to be effective as an integral part of treatment programs that aim to help prevent relapses (125). However, whether CBT has a direct benefit on craving is unclear, as results from studies to date have been inconsistent (128, 129). In a study by Ling et al. (128), no significant differences in craving were observed between patients treated with buprenorphine and standard medical management (limited counseling) or buprenorphine combined with either CBT (weekly counseling sessions, exercises, and homework) or contingency management. In contrast, more promising results were obtained from a study of patients receiving methadone maintenance therapy and combined CBT and contingency management. In this study, significantly decreased craving over the course of a 12-week period was reported, although it was limited by the lack of a control arm (129).

Mindfulness, broadly defined as a “systemic development of attention to present moment experience with an attitude of acceptance and non-judging” (130) has been linked with reduced craving in substance use disorders, albeit current evidence has limitations and displays heterogeneity between studies (130–133). In a few studies in patients receiving opioid treatment for chronic pain, mindfulness has been significantly inversely correlated with opioid craving (134–136). Mindfulness-based relapse prevention (MBRP) is a novel approach to relapse prevention (a cognitive behavioral group therapy) (137). MBRP has been shown to reduce substance use compared with treatment as usual in patients with substance use disorders in both outpatient and residential settings (137–139). MBRP may prepare patients for environmental relapse risks they encounter after completing residential treatment programs.

Although the extent to which mindfulness can influence the underlying neurobiological changes in addiction is unclear, preliminary evidence in both addiction and non-addiction settings suggests that mindfulness approaches can influence the HPA axis (140–144) and lead to structural changes, including a reduction in gray matter density in the amygdala and an increase in gray matter concentration within the hippocampus (145, 146).

## Expert Opinion

Craving is a core facet of OUD that has been shown to be associated with the risk of aberrant drug use and relapse in numerous studies. It is underpinned by neurobiological and neuroendocrine adaptations that lead to the dominance of an anti-reward pathway and a change in the set-point of hedonic tone. In daily clinical practice, craving is often still not adequately assessed; however, identifying craving triggers and evaluating the intensity and frequency of craving episodes can be an important part of the management of patients with OUD. Furthermore, craving should be considered as an important treatment target to reduce the risk of relapse and to improve patients’ quality of life. When used at optimal doses, pharmacological therapies, such as buprenorphine and methadone, can significantly reduce craving and reduce the risk of relapse. Moreover, evidence to support the benefits of non-pharmacological treatments, such as mindfulness-based therapies, as a supplementary treatment to OAT is beginning to emerge. Despite the positive impact of these treatments, some patients remain at risk of craving and, as a result, relapse to opioids. Patients may also continue to be burdened by the residual negative affect and dysphoria that result from the neurobiological adaptations associated with addiction. These patients frequently misuse other substances, such as central nervous system depressants like alcohol and cannabis, in an effort to self-medicate for the negative affect associated with the overactive amygdala. It is hoped that future studies will further our understanding of the neurobiological basis of craving and drive the development of novel therapeutic strategies along with optimized use of existing treatments in order to improve outcomes for the growing numbers of patients affected by OUD.

*Note: 32 mg dose of buprenorphine is not licensed in all countries (see local prescribing information for details).

## Author Contributions

All authors contributed to the first draft of the manuscript, critically revised subsequent drafts, and approved the final version.

## Funding

The authors declare that this study received funding from Indivior UK Ltd. Indivior funded medical writing support provided by Stephanie Carter of Spirit Medical Communications in accordance with Good Publication Practice (GPP3) guidelines. One of the authors (GS) is an employee of the funder. The funder was not otherwise involved in the study design, collection, analysis, interpretation of data, the writing of this article or the decision to submit it for publication.

## Conflict of Interest Statement

JK, HA, RM, and FN have received consultancy fees from Indivior UK Ltd. AB has received unrestricted educational grants from Schering Plough, Merck Serono, Lundbeck, and Indivior. GS is an employee of Indivior UK Ltd.
